# Scabies: A Catastrophe that Befalls Bangladesh amidst Floods

**DOI:** 10.4269/ajtmh.24-0684

**Published:** 2025-04-22

**Authors:** Sakan Binte Imran

**Affiliations:** Sir Salimullah Medical College, Dhaka, Bangladesh

Sitting in a chair beside a window in a camp, I saw traces of water everywhere. It seemed like the entire land had been submerged and was slowly receding. The crumbled houses and sheds, uprooted trees, and absence of clean water sources added more melancholy to the situation. Within a few minutes, I noticed long queues of patients assembled in front of me. Harboring both excitement and nervousness in my heart, I called the first person in line.

A man, seeming to be in his late thirties, appeared weathered and worn, with a lean, gaunt face that bore traces of suffering and hardship. He came forward. His clothes were tattered and mismatched; he wore a faded lungi that had multiple holes, patched with different fabric, and a frayed shirt that hung loosely on his frame. His shoes were scuffed and barely held together, revealing his struggle. His hair was unkempt, and his skin was weather beaten. His eyes, although tired, seemed to hold hope. I greeted him and asked him to take a seat in front of me.

Moving forward, I asked, “What are your complaints? How can I help you?” Breaking into tears, he said, “I have lost everything in this flood. These waters have engulfed my reasons to live, my shed, and my cattle.” Upon hearing his agony, I felt a deep pain in my heart.

Although I had witnessed blue clouds overcast the sky with a sound mind during early August, I met with a chilling reality upon watching the news. It showed water levels rising at an alarming rate because of heavy rainfall. Within hours, relentless monsoon rains, worsened by ongoing climate change and deforestation, unleashed a flood that submerged southern areas of Bangladesh, leaving behind a trail of distress and despair. In response to this crisis, health care professionals formed groups to set up health camps aimed at providing food and medical care to the affected population. Without hesitation, I volunteered at a camp in Alkora, Chouddagram, Cumilla. My fellow members and I had prepared boxes of medications, dry foods, and water purification tablets and set out for the flood-affected zone. This man’s pain echoed the eerie cry for help that greeted us volunteers when we arrived before dawn to camp in a school building that had been submerged in 3–4 feet of water a few days prior. Similar to that cry, I could do nothing for most of his anguish.

I tried to console him and further said, “As I can provide you with health-related solutions, do you have any complaints regarding that?” Sobbing a bit and wiping the tears from his cheeks, he said, “Yes” and showed me his hands. “I feel extreme itching in my fingers and around my belly button. The itching worsens at night, and I can hardly get any sleep because of this sheer discomfort.” I looked closely and noticed that there were multiple papules in the interdigital clefts, wrists, umbilical area, and periumbilical regions that were coupled with the complaint of worsening itching at night. Instantly, I recognized a classic textbook case of scabies.

I asked, “When did it start?” He replied, “I noticed it less than a month ago, but the itching became severe in the last few days.” I further asked, “Do your family members have the same symptoms?” He answered affirmatively. After listening to his story, I provided him with the necessary medication. Additionally, I advised him on essential do’s and don’ts. However, he expressed reluctance, stating that it was impossible to follow my guidance amidst the flooding. I urged him to try, hoping he could carry out at least some of my recommendations.

Finishing my first consultation, I moved on to my next patient. A boy in his early twenties stepped forward with a handful of empty sachets and said, “I have been using these medicines for the last few days. Can you see these?” I looked at him and said, “Kindly take a seat first. Certainly, I will examine these and provide you with an answer.” He smiled and immediately sat down. He was constantly rubbing between his fingers to the extent that they looked red, as if he had rubbed them repeatedly with intense force.

I asked, “Are you feeling any discomfort in your fingers?” He nodded affirmatively while scratching his arms. Observing the boy, I noticed he was wearing a T-shirt that appeared too small for his frame and trousers that were covered in mud, and he was barefoot. He also had scratch marks all over his arm and repeated injection marks at his cubital fossa area.

He again said, “See these medicines,” handing me the sachets. “Are they useful?”

I told him firmly, “To determine whether these medications are useful or not, I need to know your complaints regarding your health. That will allow me to make an assessment. So, what are your complaints?”

He said, “I feel very itchy between my fingers, my arms, my inner thighs, and in my axillary area. More so at night.”

Upon examination, I looked at the places that he mentioned and noticed red papules. Because of extreme scratching, some papules had burst, and a small amount of watery discharge was coming out of them. I asked, “Do you have any other problems apart from this?”

He said, “No.”

I then asked, “What are these marks on your arm showing the repeated injection marks?”

He was silent. I assured him that I was his physician and that he could be completely honest with me. This would help me provide him with a proper diagnosis and adequate treatment. He then opened up about his drug history, which he urged to keep confidential. I asked him the reason that drove him to it.

He said, “Poverty,” and he added that his life had no meaning or purpose. He saw himself as a burden to his family and society. He had no friends, and no one understood him.

Understanding the gravity of the situation, I chose to address one problem at a time. First, I asked, “Did this itching start after you began using injectable drugs?” He answered negatively, which helped me rule out folliculitis associated with drug use. I then inquired about the duration of his symptoms, his living conditions, and his family members. All of his answers pointed to one specific conclusive diagnosis: scabies. I explained his condition regarding the itching. Before prescribing him medications, I looked at the sachets he bought. Those medicines were irrelevant to the treatment of scabies. Instead, there were a few nonsteroidal anti-inflammatory drugs, antiulcerants, and antifungal medications. I advised him to discard these medicines and prescribed him the appropriate treatment of scabies. Additionally, I told him that not only he but also his family members should follow this treatment regimen to achieve a full cure.

Before ending our conversation, I advised him to visit the tertiary hospital for mental health support. Although he got agitated at first, I calmly explained that seeking help was not a sign of insanity. Everyone on this earth needs one another, and helping each other out for peace and tranquility is the best thing we can do. He gestured that he understood and promised to visit the tertiary hospital. Exchanging amicable smiles, our consultation ended.

Next, I saw a 5-year-old child in her mother’s lap. She appeared extremely irritable and hardly spoke any words, just throwing tantrums. The mother rushed and took the seat in front of me, saying, “Save my child.” I immediately went close to the child and following the “look, listen, and feel” method, assessed her. Examination revealed that she was quite eager to drink water, and the skin pinch test returned slowly, all indicating moderate dehydration because of diarrhea. However, something else caught my attention: reddish papules that seemed infected on her shins, legs, hands, and fingers. It took me no time to understand that the child had scabies with a secondary bacterial skin infection along with moderate dehydration because of diarrhea. Without any delay, I opened an intravenous channel and started fluid correction. After managing the acute emergency, I asked the mother about the reddish spots on her child’s body. She said, “She developed some water-filled blisters 2 months ago. I gave her some medicine from the pharmacy. These blisters were transparent before, but now they are looking reddish for the last week.”

I informed her that her child had scabies with moderate dehydration because of diarrhea. She was astonished as she had thought these were rashes from excessive sun exposure and had not paid much attention to them. I corrected her confusion and explained the disease, its transmission, treatment, and prevention. I provided her with the necessary treatment and precautions to follow and referred the child to the tertiary hospital for further dehydration management.

The entire patient visitation took 10 hours, with a 1.5-hour break in between. In total, I encountered around 110–115 patients that day, and shockingly, more than 75 of them were suffering from scabies, some alongside other ailments. The staggering prevalence of scabies symptoms suggested that we were on the brink of an epidemic. I was astounded by the extent of the crisis, and when I shared my observations with my teammates, they reported similar experiences.

While seeing the patients, I observed that scabies, a contagious skin disease transmitted through close skin-to-skin contact, affects common sites, like interdigital clefts, wrists, the flexor aspect of the forearm, axillary folds, nipples and areola, the umbilical and periumbilical regions, the belt line, genitalia, the perineum, the buttocks, and the inner aspect of the thighs. Most of the patients I saw presented with red papules or pustules or the presence of linear or curved skin burrows at the affected sites, which led to a clear-cut diagnosis. I provided the patients with conventional treatment of scabies, which includes treating all family members and contact individuals simultaneously using both pharmacological and nonpharmacological methods. In total, we provided consultations to over 3,500 patients in 2 days, with around 2,800 or more showing signs and symptoms of scabies.

Scabies, a burdensome yet often neglected skin disease, is closely tied to a triad: hygiene, socioeconomic status, and awareness. I clearly understood that the difficulties in maintaining a healthy lifestyle during the floods meant that more people were succumbing to the disease, whereas those already affected struggled to find cures, leading to serious complications.

Additionally, I observed that patients with better housing facilities were largely unaffected, underscoring the role of socioeconomic factors. An interesting thing I noticed was that a better understanding of contagious diseases saved some people from this disease, which highlights the importance of awareness.

I went to several different camps after this at Sonagazi Feni and Noakhali, with the intention of serving them and a motive to explore more regarding the matter previously explained. More or less, the picture was the same at those places: a massive number of patients had, along with other water-associated diseases, scabies, which is not only concerning but also, alarming.

The prevalence of scabies at a terrifying rate alongside the other diseases indicates that there is more to it. People are neither concerned nor informed regarding the consequences of poor hygiene, and that is what caused this skin disease to affect a large portion of the population.

My experience and my insights suggest that the severity of the situation is beyond description, and without immediate awareness and adequate intervention, a full-blown epidemic is on its way.

